# Animalcules in the Intestinal Canal

**Published:** 1844-06

**Authors:** 


*Animalcules in the Intestinal Canal.*—MM. Gruby and Delafond read a paper at the Academie des Sciences, on the 11th of December, on certain animalcules existing in great numbers in the stomach and intestines of herbivorous and carnivorous animals during digestion.

These physiologists, while pursuing their researches on digestion, made the discovery of numerous animalcules which are born, live, and die, in the stomachs of ruminantia—of the dog and pig, and in the large intestines of the horse. These animalcules exist in such numbers, and their presence is so constant, that they may be supposed to be of some service in the act of digestion. The following are the conclusions which may be drawn from their communication.

1. That the ruminantia have four species of living animalcules in their stomachs, during the period of digestion.

2. That the horse has seven species of living animalcules in the cæcum and dilated portion of the colon.

3. That the dog has two species of monads in the stomach.

4. The pig has only one species of animalcule in his stomach.

5. The animalcules of digeston are born, live, and swim in the acid liquor contained in the stomach.

The great number of these animalcules in the two first stomachs of ruminantia, the presence of their empty exuviæ in the third and fourth, and in the excremental matter, the considerable number of them in the cæcum and dilated colon of the horse, as also the existence of their empty exuviæ in the contracted colon and rectum, lead the authors to believe that the organic matter of these animalcules is digested in the stomach of ruminantia, and that it is absorbed in the contracted portion of the colon of the horse, and that in both cases it furnishes an animal matter for the purpose of digestion.

The conclusion to be drawn from these facts seems to be that, since in a state of nature, herbivorous animals receive only vegetable matter into their stomachs, a fifth part at least is destined to give birth to a great quantity of animals of an inferior development, who, digested in their turn, furnish animal matter for the general nourishment of herbivorous animals. This conclusion seems the more reasonable, as in the dog and pig, which are fed on animal and vegetable substances; the animalcules are smaller, less numerous, and of only one or two species.—*Prov. Med. Journ.*, from *Gaz. Medicale*, Dec. 16.

